# Redistribution of ill-defined deaths: the Scottish Burden of Disease approach: Eilidh Fletcher

**DOI:** 10.1093/eurpub/ckac129.620

**Published:** 2022-10-25

**Authors:** E Fletcher, I Grant, G Wyper, G McCartney, M Thrower, D Stockton

**Affiliations:** 1 Data Driven Innovation Directorate, Public Health Scotland, Edinburgh, UK; 2 College of Social Sciences, University of Glasgow, Glasgow, UK; 3 Place and Wellbeing Directorate, Public Health Scotland, Glasgow, UK; 4 Clinical and Protecting Health Directorate, Public Health Scotland, Glasgow, UK

## Abstract

Burden of disease (BoD) studies are an established method of quantifying health loss across - and within - a population. They aim to combine the impact of living with, and dying from, various health conditions to allow for comparability of conditions in an equitable manner. A key component of this is the calculation of the loss of years of life arising from premature death (Years of Life Lost (YLL)). Most high-income nations have robust death registration systems which ensure that deaths are routinely recorded, the causes are medically certified and the age at death is accurate. However, even in these situations the recording of ill-defined death (IDD) causes remains widespread and to some extent unavoidable, in that it is not always appropriate to undertake extensive investigation to establish an exact cause of death or the cause of death recorded does not map directly to disease groupings used routinely in BoD studies. The Scottish Burden of Disease (SBoD) uses cause of death data from the National Records of Scotland. These patient-level records include one underlying cause of death and up to 10 supplementary causes of death, all coded using ICD classifications. Around 12% of these deaths do not map directly to a BoD cause group and could therefore be considered ill-defined. The SBoD study have developed a 9-step hierarchical methodology for the redistribution of ill-defined deaths, utilising uses a mix of fixed and proportional redistribution and focusses on exploiting the data recorded on the death certificate at both an individual and population level. In this presentation we will describe the methodology used to redistribute ill-defined deaths in the Scottish study - the development, the application and the strengths and weaknesses of our approach. We will also discuss the example of COVID-19 and how competition between the underlying cause of death is likely to impact how we need to approach IDDs in the future.

